# Transition in Frailty State Among Elderly Patients After Vascular Surgery

**DOI:** 10.1007/s00268-020-05619-7

**Published:** 2020-06-03

**Authors:** Louise B. D. Banning, Linda Visser, Clark J. Zeebregts, Barbara L. van Leeuwen, Mostafa el Moumni, Robert A. Pol

**Affiliations:** 1 Department of Surgery, Division of Vascular Surgery, University of Groningen, University Medical Center Groningen, Hanzeplein 1, P.O. Box 30.001, 9700 RB Groningen, The Netherlands; 2 Department of Surgery, Division of Surgical Oncology, University of Groningen, University Medical Center Groningen, Hanzeplein 1, P.O. Box 30.001, Groningen, The Netherlands; 3 Department of Surgery, Division of Trauma Surgery, University of Groningen, University Medical Center Groningen, Hanzeplein 1, P.O. Box 30.001, Groningen, The Netherlands

## Abstract

**Background:**

Frailty in the vascular surgical ward is common and predicts poor surgical outcomes. The aim of this study was to analyze transitions in frailty state in elderly patients after vascular surgery and to evaluate influence of patient characteristics on this transition.

**Methods:**

Between 2014 and 2018, 310 patients, ≥65 years and scheduled for elective vascular surgery, were included in this cohort study. Transition in frailty state between preoperative and follow-up measurement was determined using the Groningen Frailty Indicator (GFI), a validated tool to measure frailty in vascular surgery patients. Frailty is defined as a GFI score ≥4. Patient characteristics leading to a transition in frailty state were analyzed using multivariable Cox regression analysis.

**Results:**

Mean age was 72.7 ± 5.2 years, and 74.5% were male. Mean follow-up time was 22.7 ± 9.5 months. At baseline measurement, 79 patients (25.5%) were considered frail. In total, 64 non-frail patients (20.6%) shifted to frail and 29 frail patients (9.4%) to non-frail. Frail patients with a high Charlson Comorbidity Index (HR = 0.329 (CI: 0.133–0.812), *p* = 0.016) and that underwent a major vascular intervention (HR = 0.365 (CI: 0.154–0.865), *p* = 0.022) had a significantly higher risk to remain frail after the intervention.

**Conclusions:**

The results of this study, showing that after vascular surgery almost 21% of the non-frail patients become frail, may lead to a more effective shared decision-making process when considering treatment options, by providing more insight in the postoperative frailty course of patients.

**Electronic supplementary material:**

The online version of this article (10.1007/s00268-020-05619-7) contains supplementary material, which is available to authorized users.

## Introduction

Almost 40% of people aged 65 to 74 suffer from chronic diseases, and multi-morbidity is present in 60% of people older than 75 [1]. Multi-morbidity, defined as the coexistence of at least three chronic conditions, or chronological age do not seem to be the best methods to distinguish a physically frail patient from a fit patient [2]. Elderly people frequently cope with many conditions during the last phases of their life and may suffer from handicaps and disabilities [2]. All these conditions can lead to poor outcomes after surgery or hospitalization, such as functional decline, complications and nursing home placement.

Frailty is a clinical geriatric syndrome that is frequently used to describe the most vulnerable or weakest older adults. These patients have a decreased capability to resolve homeostasis after a stressor event, which leads to an increased risk of adverse health outcomes [3]. Health professionals need to identify these patients to both anticipate and initiate preventive measures to slow progression of frailty [4].

Over the past years, various frailty measurement tools have been developed. The Fried Frailty Index, the Groningen Frailty Indicator (GFI), the G8 Questionnaire and the Edmonton Frail Scale are a few examples of instruments that contain multiple frailty domains [5–8]. These measurement tools are widely used to assess a patient’s fitness level and gain insight into the expected postoperative course [9].

Studies have already shown that frailty in the vascular surgical ward is common and that it predicts poor surgical outcome after various vascular interventions [10–14]. In these studies, however, frailty was assessed at one specific time point, assuming that it is a static state even though new insights reveal that it is rather a dynamic process that can be influenced by various factors [15–18]. One of these prior studies, that determined the changes in frailty in community-dwelling elderly people, found that after three years of follow-up, 8.2% transitioned from a pre-frail to a frail and 0.6% transitioned from a non-frail to a frail state [19]. Although this study demonstrates that frailty is dynamic, determining the degree of frailty before and after an intervention can provide information about the effect of the intervention, supporting an approach on quality of life and patient-reported outcome measures.

The aim of this study was to determine the transition of frailty state among elderly patients after elective vascular intervention and evaluate the influence of various patient characteristics and the individual frailty domains on this transition.

## Material and methods

### Study design and participants

This study is part of prospective cohort study on frail vascular surgery patients (Vascular Ageing study) at our tertiary referral teaching hospital. Between April 2014 and April 2018, 446 consecutive patients scheduled for elective vascular surgery were included in this study. Since frailty incidence is much lower in younger patients, we limited the age of participants to ≥65 years [20, 21]. Frailty was preoperatively measured (index measurement) at the outpatient clinic using the GFI. During long-term follow-up (ranging from 1 to 3 years), frailty was measured a second time for each living patient. All patients were contacted by phone. If they preferred to complete the GFI in writing, we send it by mail with an accompanying letter and consent form. Follow-up and clinical data were extracted from the Vascular Ageing database and if necessary complemented by reviewing electronic hospital registries and charts. In addition to the granted permission for the Vascular Ageing Study, the Medical Ethical Committee approved dispensation for the Dutch law on patient-based medical research (WMO) obligations (registration no. METc2017/200). Patient data were processed and electronically stored in conformity with the Declaration of Helsinki—Ethical principles for medical research involving human subjects [22]. Data were analyzed anonymously.

### Assessment of frailty

Frailty was measured twice using the GFI, whose feasibility, sensitivity and specificity had previously been tested in various studies, such as a study on postoperative delirium after vascular surgery [8, 23, 24]. The GFI is classified into eight separate groups, divided over 15 questions, consistent with the domains of functioning: mobility (0–4 points), visual functioning (0–1 point), auditory functioning (0–1 point), nutrition (0–1 point), comorbidity (0–1 point), cognition (0–1 point), psychosocial aspects (0–5 point) and physical fitness (0–1 point), with a total end score ranging from 0 to 15. Based on previous publications, frailty was defined as GFI score ≥4 [6, 25].

### Transition in frailty state

To determine the primary outcome, the transition in frailty state between index and follow-up measurement, we calculated for each patient if their frailty score (GFI ≥4) remained unchanged, shifted from frail to non-frail or from non-frail to frail. Next, we analyzed which patient characteristics were associated with this transition. To determine the change in the separate GFI domains, a sub-analysis (transition from 0 to ≥1 point, no change or transition from ≥1 to 0 points) per GFI domain was performed.

### Baseline variables

Preoperatively and intraoperatively collected data included age, gender, body mass index (BMI), smoking (never/quit/current), hypertension, comorbidities, type of surgery and American Society of Anesthesiologists’ physical status classification system score (ASA score). BMI was calculated as weight/height^2^. Comorbidity was assessed using the Charlson Comorbidity Index, a weighted score that predicts the one-year mortality of a patient based on coexisting medical conditions and age [26]. To determine the Charlson Comorbidity Index, we used the calculator developed by Hall et al. [27] Endovascular peripheral interventions were classified as simple interventions (angioplasty and single stents). Endovascular aortic interventions included endovascular aneurysm repair (EVAR), thoracic endovascular aneurysm repair (TEVAR), fenestrated endovascular aneurysm repair (FEVAR) and branched endovascular aneurysm repair (BEVAR). Abdominal interventions were defined as open reconstructions through a midline or oblique trans- or retroperitoneal incision. Major vascular surgery included all the above-mentioned interventions, excluding shunt, endovascular peripheral and miscellaneous (minor nectrotectomies and minor amputations) interventions.

Postoperatively collected data included complications, intensive care unit (ICU) admission during hospital stay and admission and surgery during follow-up. Postoperative complications were registered and analyzed using the Comprehensive Complication Index, a tool that summarizes all postoperative complications by severity according to the Clavien–Dindo classification of surgical complications [28, 29]. Follow-up time was defined as the time (months) between the index and follow-up measurement of the GFI.

### Statistical analysis

When normal distribution was assumed with help from a Q-Q plot and a histogram, continuous variables were presented as mean ± standard deviation (SD). For a skewed distribution, data were presented as median ± interquartile range (IQR). Categorical variables were presented as numbers and percentages. We performed an analysis of the association between transition in frailty state and the earlier mentioned patient characteristics. Differences between continuous variables were tested with the analysis of variance (ANOVA) test for normal distribution and the Mann–Whitney U test for skewed distribution. Differences between categorical variables were tested with the Chi-squared test. Subsequently, multivariable analysis was performed using Cox regression with transitions from non-frail to frail and frail to non-frail as dependent variables and age, gender, smoking, BMI, comorbidities and type of surgery as independent variables. These variables were selected based on the literature and univariable analysis (*P* < 0.30). Estimates in mean differences were reported with corresponding 95% confidence intervals. A *p* value ≤ 0.05 was considered statistically significant. Statistical analyses were performed with the Statistical Package for the Social Sciences (IBM© SPSS Statistics© Version 23).

## Results

### Baseline characteristics

A total of 65 patients died during follow-up, and 71 patients declined or did not respond, resulting in 310 patients that formed the basis for this analysis. In total, 129 patients were considered frail (28.9%), of which 79 patients (25.4%) in the study cohort and 50 patients (36.7%) in the excluded cohort. Mean age of the study cohort was 72.7 ± 5.2 years, with 231 (74.5%) being male (Table 1). Mean BMI was 26.9 ± 4.0. Most common procedure was an endovascular aortic intervention (35.8%). Mean follow-up time was 22.7 ± 9.5 months. During follow-up, 35.2% of patients were admitted to the hospital at least once and 24.8% required surgery.

**Table 1:** Baseline characteristics

Variable	Total (n = 310 patients)
Age (years)	72.7 ± 5.2
Gender	
Male	231 (74.5%)
Female	79 (25.5%)
Smoking^a^	185 (59.7%)
BMI	26.9 ± 4.0
Comorbidities^b^	5.1 ± 1.6
Type of surgery	
Endovascular peripheral	42 (13.5%)
Endovascular aortic	111 (35.8%)
Peripheral bypass	33 (10.6%)
Carotid	78 (25.2%)
Abdominal	30 (9.7%)
Amputation below the knee	2 (0.6%)
Miscellaneous	14 (4.5%)
ASA^c^ score, median (IQR)	3 (2–3)
Length of hospital stay (days), median (IQR)	4 (4–7)
Admittance to ICU	77 (24.8%)
Postoperative complications^d^, median (IQR)	0 (0–9)
30-day readmission	15 (4.8%)
Follow-up time (months)	22.7 ± 9.5
Admission during follow-up	109 (35.2%)
Surgery during follow-up	77 (24.8%)

Data are presented as *N* (%) or mean ± SD unless stated otherwise

^a^History of smoking

^b^According to the Charlson Comorbidity Index, a weighted index that predicts one-year mortality by measuring the burden of comorbidities (range 0–19)

^c^American Society of Anesthesiologists’ score, categorizes fitness of patients prior to surgery (range 1–5)

^d^According to the Comprehensive Complication Index, which takes all complications after a procedure and their respective severity into account (range 0–100)

### Transition in frailty state: descriptive

**Fig. 1:**
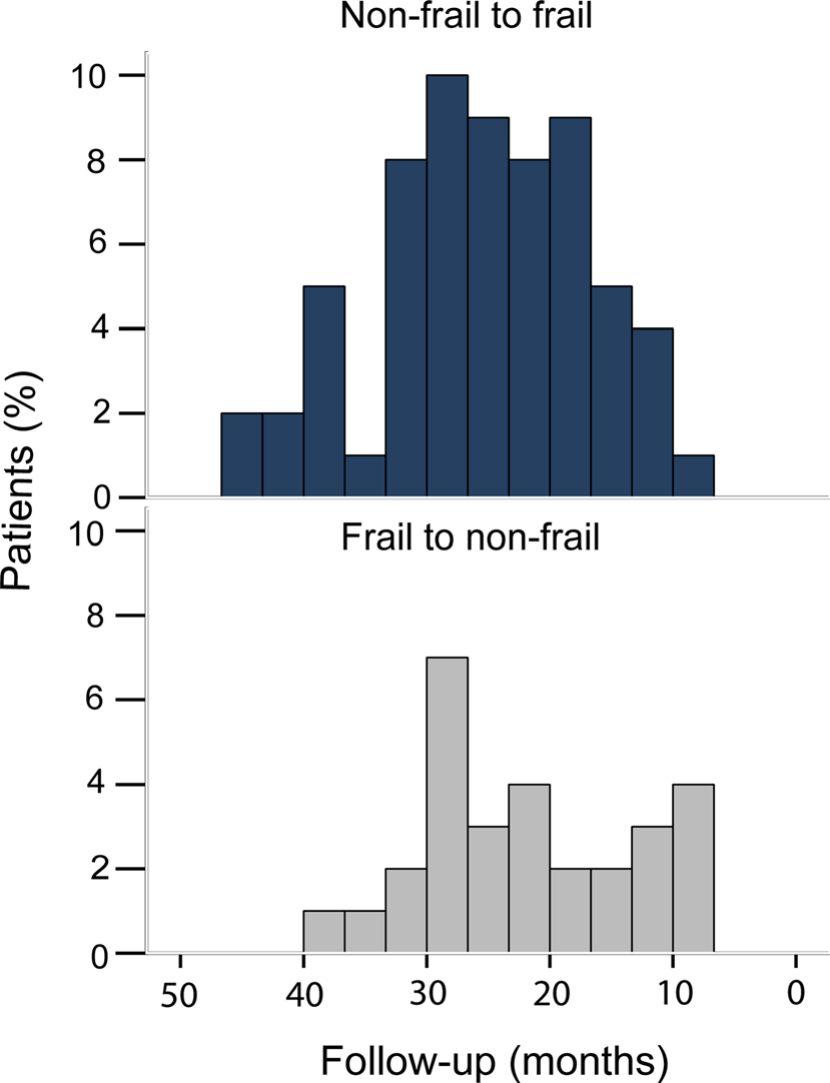
Transition in frailty state percentage of patients that transitioned in frailty state during follow-up (months)

During follow-up, 167 patients (53.9%) remained non-frail and 50 patients (16.1%) remained frail (Table 2). Sixty-four patients (20.6%) shifted from non-frail to frail and 29 patients (9.4%) from frail to non-frail. As shown in Fig. 1, the number of non-frail patients that shifted to frail increased over time.

**Table 2:** Transition in frailty state

Total (*n* = 310 patients)			
Frail^a^ preoperative	79 (25.5%)		
Frail^a^ during follow-up	114 (36.8%)		
Transition in frailty state	From	To	
		Non-frail	Frail
	Non-frail	167 (53.9%)	64 (20.6%)
	Frail	29 (9.4%)	50 (16.1%)

^a^According to the GFI cut-off of ≥4

### Transition in frailty state: association with patient characteristics

Table 3 displays the association of the patient characteristics with transitions in frailty state. Age did not differ significantly between the three groups (*p* = 0.579). A higher percentage of patients in the frail to non-frail group smoked compared to the non-frail to frail group (54.1% vs. 72.4%); however, this did not reach statistical significance (*p* = 0.215). There was no statistical difference in follow-up time between the groups (*p* = 0.249). The non-frail patients that transitioned to frail had a significantly higher Charlson Comorbidity Index than the frail patients that transitioned to non-frail (5.2 ± 1.5 vs. 4.3 ± 1.4, p = 0.011). Patients (*N* = 75) that underwent revascularization had a higher share in the frail to non-frail group than in the non-frail to frail group (34.5% vs. 21.9%). In Table 4, the multivariable Cox regression analysis is shown. Frail patients with a high Charlson Comorbidity Index (HR = 0.579 (CI: 0.432–0.776), *p* < 0.001) and patients that underwent a major vascular intervention (HR = 0.417 (CI: 0.198–0.881), *p* = 0.022) had a significantly higher risk to remain frail.

**Table 3:** Patient characteristics associated with transition in frailty state

Variable	Non-frail to frail (*n* = 64)	No change (*n* = 217)	Frail to non-frail (*n* = 29)	*p* value^a^
Age	73.3 ± 5.8	72.6 ± 5.1	72.4 ± 5.0	0.579
Gender				
Male	46 (71.6%)	165 (76.0%)	20 (69.0%)	0.616
Female	18 (28.1%)	52 (24.0%)	9 (31.0%)	
Smoking^b^	33 (54.1%)	132 (63.2%)	21 (72.4%)	0.215
BMI	27.6 ± 3.5	26.8 ± 4.1	26.0 ± 3.9	0.228
Comorbidities^c^	5.2 ± 1.5	5.2 ± 1.7	4.3 ± 1.4	0.011
Type of surgery				
Endovascular peripheral	7 (10.9%)	31 (14.3%)	4 (13.8%)	0.837
Endovascular aortic	23 (35.9%)	78 (35.9%)	10 (34.5%)	
Peripheral bypass	7 (10.9%)	20 (9.2%)	6 (20.7%)	
Carotid	18 (28.1%)	54 (24.9%)	6 (20.7%)	
Abdominal	5 (7.8%)	24 (11.1%)	1 (3.4%)	
Amputation below the knee	0 (0.0%)	2 (0.9%)	0 (0.0%)	
Miscellaneous	4 (6.3%)	8 (3.7%)	2 (6.9%)	
ASA^d^ score, median (IQR)	3 (2–3)	2 (2–3)	2 (2–3)	0.349
Length of hospital stay (days), median (IQR)	4 (3–7)	4 (4–7)	5 (3–8)	0.455
Admittance to ICU	15 (23.4%)	57 (26.3%)	5 (17.2%)	0.549
Postoperative complications^d^, median (IQR^g^)	0 (0–11.3)	0 (0–8.7)	0 (0–8.7)	0.992
30-day readmission	3 (4.7%)	12 (5.7%)	0 (0.0%)	0.440
Follow-up time (months)	24.5 ± 8.6	22.3 ± 98	22.0 ± 9.0	0.249
Admission during follow-up	25 (39.1%)	77 (35.5%)	7 (24.1%)	0.371
Surgery during follow-up	17 (26.6%)	55 (25.3%)	5 (17.2%)	0.598

Data are presented as *N* (%) or mean ± SD unless stated otherwise

^a^*P* values ≤ 0.05 were considered statistically significant

^b^History of smoking

^c^According to the Charlson Comorbidity Index, a weighted index that predicts one-year mortality by measuring the burden of comorbidities (range 0–19)

^d^According to the Comprehensive Complication Index, which takes all complications after a procedure and their respective severity into account (range 0–100)

**Table 4:** Cox regression analysis of factors associated with transition in frailty state

Variable	Non-frail to frail (*n* = 64) Hazard ratio (95% CI)	*P* value	Frail to non-frail (*n* = 29) Hazard ratio (95% CI)	*p* value^a^
Age	1.014 (0.963–1.068)	0.600	1.048 (0.973–1.130)	0.215
Gender(female)	1.167 (0.660–2.063)	0.595		
Smoking^b^	0.878 (0.514–1.499)	0.634		
BMI	1.045 (0.978–1.117)	0.191		
Comorbidities^c^	0.934 (0.777–1.123)	0.467	0.579 (0.432–0.776)	<0.001
Type of surgery (major vascular interventions^d^)	0.723 (0.407–1.286)	0.270	0.417 (0.198–0.881)	0.022

^a^*P* values ≤ 0.05 were considered statistically significant

^b^History of smoking

^c^According to the Charlson Comorbidity Index, a weighted index that predicts one-year mortality by measuring the burden of comorbidities (range 0–19)

^d^Excl. shunt, endovascular peripheral and miscellaneous (minor nectrotectomies and minor amputations) interventions

### Change per GFI domain

**Fig. 2:**
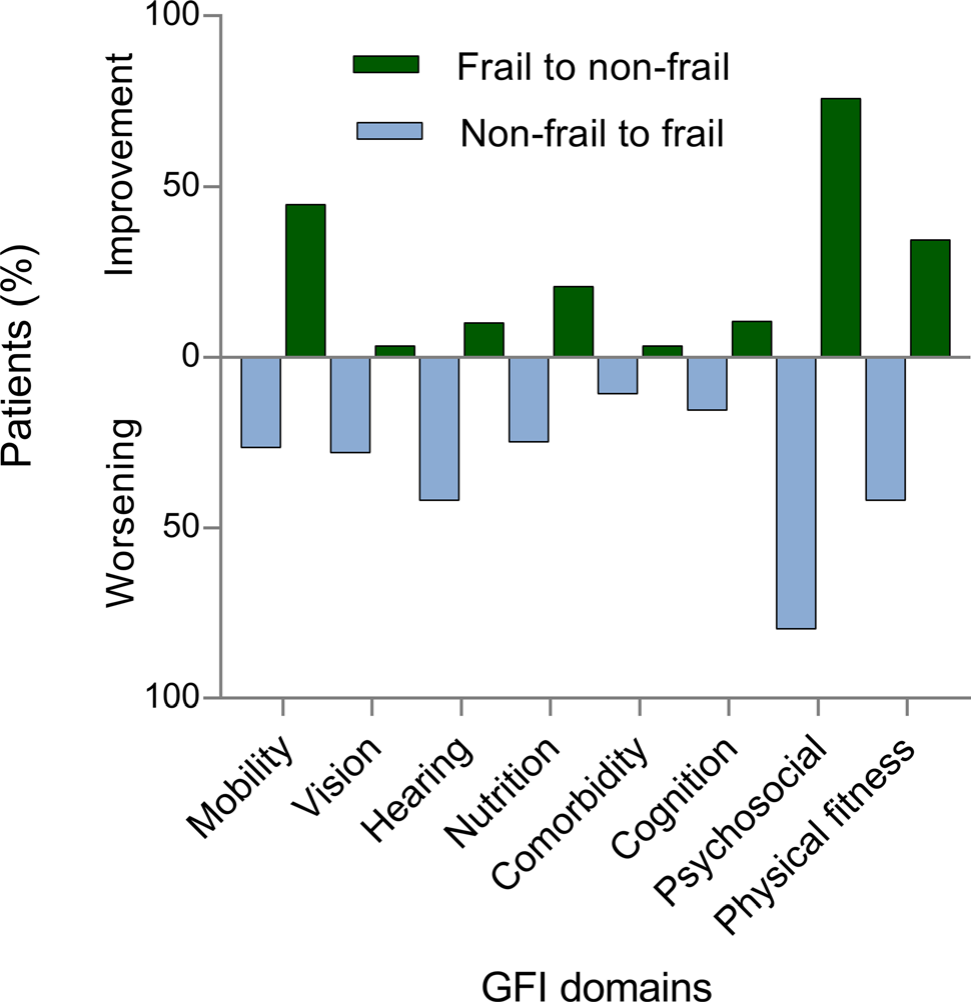
Change in GFI score, per domain change in Groningen Frailty Indicator (GFI) score, stratified in patients (%) that transitioned from frail to non-frail (improvement in GFI score) and from non-frail to frail (worsening in GFI score), per domain

Changes in GFI score, calculated per domain, are shown in Fig. 2 and Supplemental Table 1. The domains *mobility*, *psychosocial* and *physical fitness* changed most in frail patients that transitioned to non-frail. As seen in Fig. 2, 44.8%, 75.9% and 34.5% of these patients, respectively, had a decreased score (“improvement”) in these domains during follow-up. For non-frail patients that transitioned to frail, the domains *hearing* (42.2%), *psychosocial* (75.9%) and *physical fitness* (42.2%) contributed most to an increased score (“worsening”) during follow-up.

## Discussion

This study shows that frailty is a dynamic condition in elderly patients undergoing an elective vascular intervention. After a mean follow-up of 23 months, almost 21% of the non-frail patients transitioned to frail and 9% from frail to non-frail. Frail patients with more comorbidities and undergoing major vascular interventions were less likely to be non-frail after surgery. As frailty is a multidimensional syndrome, the domains that determine degree and type of frailty are an important part of the personalized approach that is necessary for the treatment or prevention of frailty-related complications. In our study, the domains *hearing impairment, diminished psychosocial functioning* and *physical fitness* contributed most to an increase in frailty during follow-up.

The present study is unique in its approach to both frailty composition and the changes that occur after vascular surgery. Although the concept has previously been demonstrated in kidney transplant recipients, this method of analysis has not been applied to vascular surgery patients. In that particular kidney transplant study, 74% of kidney transplant recipients who were frail at the time of the transplantation transitioned to intermediately frail or non-frail after three months [18]. Of those patients, 47% improved in strength, 55% in physical activity and 19% in gait speed. Although our study certainly has parallels, the effect of a kidney transplantation, compared to the continuation of dialysis, on postoperative cognitive and physical functioning cannot be compared to end-stage vascular patients. And yet, together with our results it further stresses the dynamic nature of frailty.

The natural course of frailty in community-dwelling individual patients aged 70 years and older has been studied before. After three years of follow-up, 8% transitioned from pre-frail to frail and 1% transitioned from non-frail to frail [19]. Because in our study almost 21% of the patients transitioned from non-frail to frail after intervention, medical practitioners should keep in mind that vascular interventions might not lead to improvement of frailty, or even quality of life and survival [30].

Frail patients with comorbidities had a significantly higher risk of remaining frail after surgery, which further strengthens the mutual ratio [31]. Additionally, this was also the case for frail patients that underwent a major vascular surgery. A possible explanation might be that these frail patients had a longer hospital length of stay and were more prone to develop postoperative complications, leading to an inability to become non-frail after surgery.

To determine frailty in this study, we used the GFI, a short and simple questionnaire compared to many other available tools, making it suitable to use at a busy outpatient clinic [32]. In addition, some of the available tools only consider the cognitive or functional domain, whereas multi-domain tools like the GFI take all the domains of frailty into account [33]. Because of these essential differences in frailty instruments, it is difficult to compare these tools with each other, especially since some domains of frailty have a more powerful effect on outcome than others [34].

Since we used a multi-domain tool like the GFI, a detailed analysis of the individual frailty components was possible. Around 30% of persons aged ≥ 65 suffer from hearing loss, which might explain the increase in the domain *hearing impairment*.[35] Additionally, it seems to be consistent with other research which showed that hearing impairment is a main determinant of deterioration in frailty [36]. Next to hearing impairment, a diminished psychosocial functioning had a major contribution. This finding is in accordance with prior studies indicating that depressive symptoms are present in 15–20% of the elderly and demonstrating the close association between multi-morbidity and depressive symptoms [37–39]. More attention should therefore be given to the assessment of depressive symptoms in a vulnerable group such as elderly, hospitalized patients.

Patient mobility, according to the GFI, did not change much during follow-up. Our hypothesis was that revascularization would improve their ability to perform the tasks defining this domain, which is in accordance with our finding that the share of frail revascularized patients that became non-frail was relatively high [40, 41]. On the other hand, the percentage of patients whose physical fitness worsened was higher than the percentage whose physical fitness improved—a finding that was previously reported [42]. Presumably, physical fitness is more a subjective feeling and might not be related to the degree of mobility. Furthermore, it was quite unexpected that patients’ cognition showed the least change compared to the other domains, since the prevalence of cognitive impairment in older vascular surgical patients is high and hospitalization is a known risk factor [43]. A possible explanation is that the GFI only assesses memory problems, and not executive dysfunction and visuospatial deficits.

Preoperative frailty in vascular surgery patients, including cognitive impairment, predicts a variability of adverse short-term outcomes like prolonged hospital length of stay, discharge to a care facility and postoperative delirium, as well as poor mid-term outcomes like 12-month mortality and higher readmission rates [23, 43–45]. It is therefore important to preoperatively identify patients at risk in order to implement personalized preoperative care, such as preventive nursing interventions, including early mobilization, oral and nutritional assistance (e.g., oral hygiene and postoperative dietary education) and orienting communication (e.g., orientation and engaged conversation) [46]. Recent studies demonstrated that prehabilitation in frail patients, consisting of physical exercises, can be helpful in decreasing the amount of adverse postoperative outcomes, like delirium and hospital length of stay [47, 48]. Possibly, prehabilitation can also help frail patients to transition to a non-frail state after surgery [49]. Therefore, the results of this study may lead to a more effective shared decision-making process when considering treatment options, by providing more insight in the postoperative frailty course of the patient [50].

Several limitations of our study need to be addressed. First, obtaining the GFI by telephone creates the risk of bias. Patients may not be giving all the information correctly because they have to answer immediately. By offering the alternative to send the questionnaire by mail we tried to limit this risk. Our method led to a response rate of 81%, which seems sufficient to create a representative sample. Second, only 310 of the 446 patients of this study were analyzed due to death or failure to respond. Although transition of frailty state of those patients is unknown, there were more patients that were preoperatively frail in that group (39%) than in the analyzed group (26%). This sample bias may have led to an underestimation of the proportion of patients that transitioned to another frailty state. Third, in this study we deliberately included elective surgical procedures only. Transitions in frailty state could have been more substantial when including acute vascular procedures. Lastly, we measured the transition in frailty only once. Changes in GFI scores that occurred over shorter periods of time may have been missed.

In conclusion, we showed that frailty is dynamic among vascular surgery patients and most likely influenced by the surgical intervention. These results can be used to support expectations in select groups of elderly patients and help in the decision-making process when considering treatment options. Further research is necessary to provide more insight into the complete transition pattern of frailty after vascular surgery with emphasis on quality of life, nutritional state and patient survival.

## Electronic supplementary material

Below is the link to the electronic supplementary material.

Supplementary file1 (DOCX 13 kb)
