# Supplementary material for: Transition in Frailty State Among Elderly Patients After Vascular Surgery
Source: World J Surg. 2020 Jun 3;44(10):3564–72. doi: 10.1007/s00268-020-05619-7 (PMC7458900; doi:10.1007/s00268-020-05619-7)
Supplement: Supplementary file 1 — Supplementary file1 (DOCX 13 kb) [file 268_2020_5619_MOESM1_ESM.docx]

**Supplemental data**

**Supplemental table 1.** Transition in GFI score during follow-up, per domain

| **GFI domain** | **Transition from 0 to ≥1 (“worsening”)** | **Transition from ≥1 to 0 (“improvement”)** |
| --- | --- | --- |
| **Mobility**  *Non-frail to frail (n=64)*  *Frail to non-frail (n=29)*  **Total (n=310)** | 17 (26.7%) 0 (0.0%)  33 (10.6%) | 1 (1.6%) 13 (44.8%)  30 (9.7%) |
| **Vision**  *Non-frail to frail (n=64)*  *Frail to non-frail (n=29)*  **Total (n=310)** | 18 (28.1%)  0 (0.0%)  42 (13.5%) | 4 (6.3%)  1 (3.4%)  7 (2.3%) |
| **Hearing**  *Non-frail to frail (n=64)*  *Frail to non-frail (n=29)*  **Total (n=310)** | 27 (42.2%)  0 (0.0%)  64 (20.8%) | 2 (3.2%)  3 (10.3%)  14 (4.6%) |
| **Nutrition**  *Non-frail to frail (n=64)*  *Frail to non-frail (n=29)*  **Total (n=310)** | 16 (25.0%)  0 (0.0%)  33 (10.7%) | 1 (1.6%)  6 (20.7%)  22 (7.1%) |
| **Co-morbidity**  *Non-frail to frail (n=64)*  *Frail to non-frail (n=29)*  **Total (n=310)** | 7 (10.9%)  3 (10.3%)  39 (12.7%) | 4 (6.3%)  1 (3.4%)  23 (7.5%) |
| **Cognition**  *Non-frail to frail (n=64)*  *Frail to non-frail (n=29)*  **Total (n=310)** | 10 (15.6%)  1 (3.6%)  23 (7.4%) | 1 (1.6%)  3 (10.7%)  13 (4.2%) |
| **Psychosocial**  *Non-frail to frail (n=64)*  *Frail to non-frail (n=29)*  **Total (n=310)** | 51 (79.7%)  1 (3.4%)  100 (32.3%) | 1 (1.6%)  22 (75.9%)  51 (16.5%) |
| **Physical fitness**  *Non-frail to frail (n=64)*  *Frail to non-frail (n=29)*  **Total (n=310)** | 27 (42.2%)  3 (10.3%)  61 (20.2%) | 5 (7.8%)  10 (34.5%)  64 (21.2%) |
